# Single swim sessions in *C. elegans* induce key features of mammalian exercise

**DOI:** 10.1186/s12915-017-0368-4

**Published:** 2017-04-10

**Authors:** Ricardo Laranjeiro, Girish Harinath, Daniel Burke, Bart P. Braeckman, Monica Driscoll

**Affiliations:** 1 Department of Molecular Biology and Biochemistry, Nelson Biological Laboratories, Rutgers, The State University of New Jersey, Piscataway, NJ USA; 2 Department of Biology, Ghent University, Ghent, Belgium

**Keywords:** Exercise, *C. elegans*, Muscle, Oxidative stress, Metabolism

## Abstract

**Background:**

Exercise exerts remarkably powerful effects on metabolism and health, with anti-disease and anti-aging outcomes. Pharmacological manipulation of exercise benefit circuits might improve the health of the sedentary and the aging populations. Still, how exercised muscle signals to induce system-wide health improvement remains poorly understood. With a long-term interest in interventions that promote animal-wide health improvement, we sought to define exercise options for *Caenorhabditis elegans*.

**Results:**

Here, we report on the impact of single swim sessions on *C. elegans* physiology. We used microcalorimetry to show that *C. elegans* swimming has a greater energy cost than crawling. Animals that swam continuously for 90 min specifically consumed muscle fat supplies and exhibited post-swim locomotory fatigue, with both muscle fat depletion and fatigue indicators recovering within 1 hour of exercise cessation. Quantitative polymerase chain reaction (qPCR) transcript analyses also suggested an increase in fat metabolism during the swim, followed by the downregulation of specific carbohydrate metabolism transcripts in the hours post-exercise. During a 90 min swim, muscle mitochondria matrix environments became more oxidized, as visualized by a localized mitochondrial reduction-oxidation-sensitive green fluorescent protein reporter. qPCR data supported specific transcriptional changes in oxidative stress defense genes during and immediately after a swim. Consistent with potential antioxidant defense induction, we found that a single swim session sufficed to confer protection against juglone-induced oxidative stress inflicted 4 hours post-exercise.

**Conclusions:**

In addition to showing that even a single swim exercise bout confers physiological changes that increase robustness, our data reveal that acute swimming-induced changes share common features with some acute exercise responses reported in humans. Overall, our data validate an easily implemented swim experience as *C. elegans* exercise, setting the foundation for exploiting the experimental advantages of this model to genetically or pharmacologically identify the exercise-associated molecules and signaling pathways that confer system-wide health benefits.

**Electronic supplementary material:**

The online version of this article (doi:10.1186/s12915-017-0368-4) contains supplementary material, which is available to authorized users.

## Background

Human exercise has been documented to protect against diabetes, cancer, cardiovascular disease, and age-associated decline in muscle, immune, and cognitive function [1, 2]. In fact, regular physical exercise is the most powerful intervention known to confer positive impacts on health and aging [3]. Still, the molecular and cellular mechanisms by which exercise brings about organism-wide benefits remain poorly understood. Part of this gap in knowledge may be due to a paucity of short-lived genetic models that can be exploited to address fundamental questions on the exercise benefits for whole-animal healthy aging. Importantly, powerful exercise training paradigms based on climbing activity have been described in *Drosophila* [4, 5]. Exercise adaptations in flies include improvements in mobility and cardiac performance, increased mitochondrial activity and quality, and increased lipolysis [4–6]. Although these studies support that exercise benefits are conserved from invertebrates to humans, the area of invertebrate exercise genetics remains strikingly underdeveloped.

The nematode *Caenorhabditis elegans* holds considerable promise as an informative exercise model given its unique advantages: a transparent body that allows live imaging of multiple tissues, single cells, and even cellular organelles; a short lifespan with a multitude of characterized biological aging markers that would allow exercise outcomes to be studied over the entire adult life of the organism; and an array of available genetic tools that would allow the molecular pathways involved in exercise benefits to be delineated. Importantly, features of *C. elegans* musculature are conserved in humans. *C. elegans* has both striated and non-striated muscles. The 95 body wall muscle cells, arranged in four longitudinal bundles, form the striated muscle system in *C. elegans*. Body wall muscle is essential for locomotion in *C. elegans* and is the functional equivalent of vertebrate skeletal muscle. The basic functional unit of striated muscles, the sarcomere, is highly conserved from nematodes to mammals in terms of the overall structure, composition, and function [7–9]. In addition to the fully characterized musculature, a completely mapped nervous system, formed by 302 neurons, could facilitate for the first time an analysis of the organism-wide effects of exercise at the single neuron level, over adult life.

Here, we establish *C. elegans* swimming as a physical exercise that exhibits key features of mammalian exercise: increased muscular metabolic rate, post-exercise locomotory fatigue, muscle mitochondrial oxidation followed by a specific oxidative stress response, and changes in carbohydrate and fat metabolism. The analyses we present here focus on the essential initial documentation of the acute response to a single swim bout, which constitutes the first step toward fully characterizing the physiological changes promoted by exercise in *C. elegans*. Many mammalian exercise adaptations derive from the cumulative effect of single training sessions [3, 10, 11]. Thus, understanding the acute response phase to exercise in *C. elegans* establishes the critical groundwork for future studies focused on long-term exercise adaptations that confer trans-tissue health and counter the natural aging process.

## Results

### Swimming is more energetically demanding than crawling for *C. elegans*

*C. elegans* locomotor patterns are modulated by their physical environment, with crawling the behavioral response on the surface of firm substrates like agar, and swimming the behavior adopted in liquids. The increased activity of *C. elegans* in a liquid environment, revealed by a higher frequency undulatory behavior [12–14], might suggest that this locomotion mode requires more energy than crawling. However, the mechanical resistance an animal encounters when crawling on an agar surface can be up to 10,000-fold larger than that encountered when swimming in a low viscosity liquid [15, 16]. Given the considerable differences in swim versus crawl behavior, we sought to resolve the question of which locomotor behavior is more energetically demanding by directly measuring the metabolic rate of *C. elegans* under both conditions and calculating the energy cost of the swim and the crawl.

**Fig. 1:**
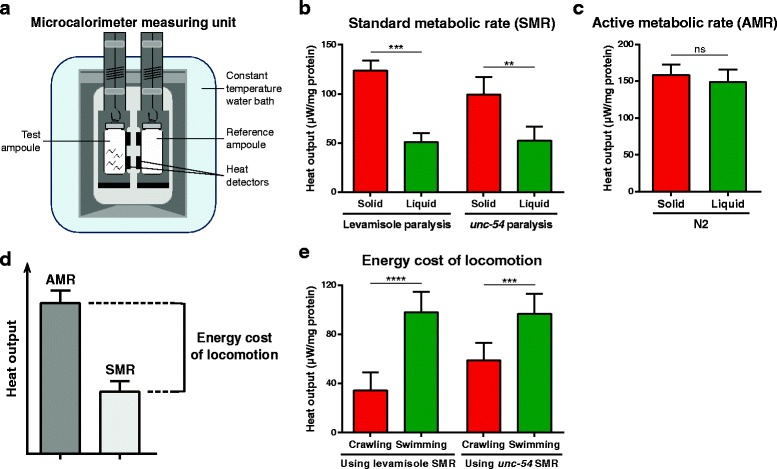
Swimming is more energetically demanding than crawling for *C. elegans*. **a** A microcalorimeter measuring unit composed of a reference ampoule (without animals) and a test ampoule (with *C. elegans*) within a precisely temperature-regulated water bath. Heat flows are monitored by super-sensitive heat detectors. **b** Microcalorimetry measurements of standard metabolic rate (*SMR*, metabolic rate at rest) at 20 °C on a solid (nematode growth medium (NGM) agar) or in a liquid (M9 buffer) environment. We immobilized animals for SMR measurements by levamisole-induced paralysis for N2 animals (*n* = 4 independent trials) or by genetic paralysis of *unc-54* mutants deficient in a major muscle myosin (*n* = 5 independent trials), and measured heat output normalized to the total amount of protein in each sample. **c** Microcalorimetry measurements of active metabolic rate (*AMR*) at 20 °C on a solid (NGM agar) or in a liquid (M9 buffer) environment by N2 animals (*n* = 7 independent trials). Heat output was normalized to the total amount of protein in each sample. Because *unc-54* mutants cannot move, their AMR cannot be measured. **d** Calculating the energy cost of locomotion. The energy cost of crawling or swimming equals the difference between the AMR and the SMR on a solid or in a liquid environment, respectively. **e** Energy cost of both locomotion forms calculated based on the microcalorimetry measures presented in (b) and (c). Note that when using *unc-54* SMR to calculate the energy cost of locomotion, we compared it to N2 AMR, and the genetic background of these two strains might differ slightly. Statistical significance was determined by paired two-tailed Student’s *t* test. **P < 0.01; ***P < 0.001; ****P < 0.0001. *ns* non-significant

Microcalorimetry directly assays the heat produced by live *C. elegans* and measures the total metabolic rate (the heat generated includes that produced by both aerobic and anaerobic metabolism) [17] (Fig. 1a). We first measured the standard metabolic rate (SMR) at 20 °C, which corresponds to the energy consumed by *C. elegans* when there is no locomotion. We immobilized animals either by levamisole-induced paralysis of wild-type N2 animals or by the genetic paralysis of *unc-54* mutants deficient in a muscle myosin heavy chain. Microcalorimetry measures for both chemical and genetic immobilization showed that stationary animals spent significantly less energy in a liquid environment than on a solid environment (Fig. 1b), possibly due to differences in oxygen availability on agar versus liquid. We next measured the active metabolic rate (AMR), which corresponds to the total energy consumed during a unit time of locomotion. We found that the AMR was similar for N2 crawling on an agar surface and swimming in M9 buffer (Fig. 1c). We then calculated the energy cost of both locomotion forms by subtracting the measured SMR from the AMR (Fig. 1d). Our energy cost calculations revealed that swimming was a more energetically demanding locomotion mode for *C. elegans* than crawling, requiring enhanced energy as compared to the cognate immobilized baseline (Fig. 1e). The *C. elegans* body wall muscle that mediates locomotion is likely the focus for most energy expenditure during swimming. Given the mammalian exercise definition as “any planned, structured, and repetitive bodily movement produced by skeletal muscles that results in energy expenditure [18]”, our findings suggest that swimming can be considered as exercise for the nematode *C. elegans*.

### *C. elegans* get tired after acute swim exercise

**Fig. 2:**
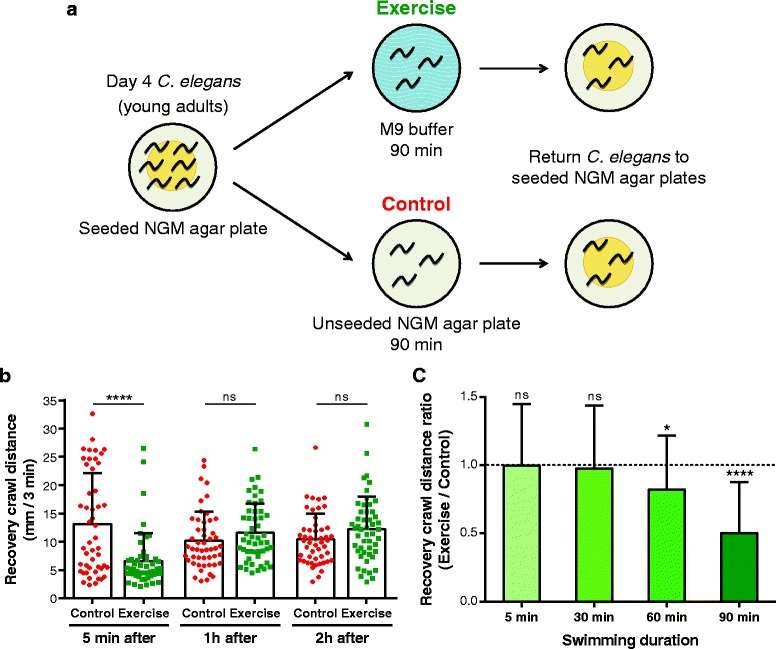
*C. elegans* get tired after acute swim exercise. **a** The acute swim exercise (M9 buffer) and control (unseeded plate crawl) protocol for *C. elegans*. **b** Crawling distance traveled by N2 animals at different time points after a 90 min swim exercise. Each point represents a single animal (*n* = 49–50 animals). **c** Crawling distance ratio of exercise to control N2 animals 5 min after a swim exercise of displayed durations (*n* = 40–50 animals). Unpaired two-tailed Student’s *t* test was used in (b) and (c) to compare crawling distances of control versus exercise animals. *P < 0.05; ****P < 0.0001. *NGM* nematode growth medium, *ns* non-significant

Young adult *C. elegans* swim continuously in M9 buffer for just over 90 min before entering an episodic phase during which periods of active swimming alternate with periods of quiescence [19]. For that reason, we decided to adopt an acute exercise protocol in which young adult *C. elegans* continuously swam in M9 buffer up to the 90 min transition point and were then returned to a bacterially seeded nematode growth medium (NGM) agar plate. We transferred crawling control animals for the same 90 min to an unseeded NGM agar plate (Fig. 2a). This experimental design guaranteed that any differences observed between exercised and control animals were not due to differences in food availability or to the rough handling associated with pick-mediated transfer of animals. For all the experiments presented here, samples were collected either immediately before exercise or at different time points post-exercise.

We observed the crawling behavior of animals upon return to food-containing plates after a 90 min swim. We measured the locomotion on plates by the track length left on the bacterial lawns to show that animals that had swum for 90 min initially moved less than their non-swim siblings (Fig. 2b). Our quantification 5 min immediately after the end of the swim exercise revealed that exercised animals crawled less than half the distance covered by the control animals when returned to the seeded NGM agar plates. Notably, however, exercised animals recovered to normal movement levels within 1 hour, as we found no difference in crawling distance between exercised and non-exercised controls at 1 and 2 hours post-exercise (Fig. 2b).

To determine if the reduction in locomotion distance might be attributed to a change in the physical environment (liquid to solid), we exercised *C. elegans* for different periods of time. When animals swam in M9 buffer for 5 min or 30 min, we found no reduction in the crawling distance after animals were returned to seeded plates (Fig. 2c), showing that this temporary slow-down phenotype is not a consequence of the transition from a liquid to a solid locomotory mode. In fact, we observed the first signs of tiring and reduced crawling ability only after a 60 min swim exercise, which became more pronounced with the 90 min exercise period (Fig. 2c). Our results reveal that a long swim exercise can induce locomotory fatigue as measured shortly after exercise completion. The fact that *C. elegans* get tired after an acute swim period further supports that swimming is more energetically demanding than crawling.

### *C. elegans* swim exercise is associated with an increase in mitochondrial oxidative stress

**Fig. 3:**
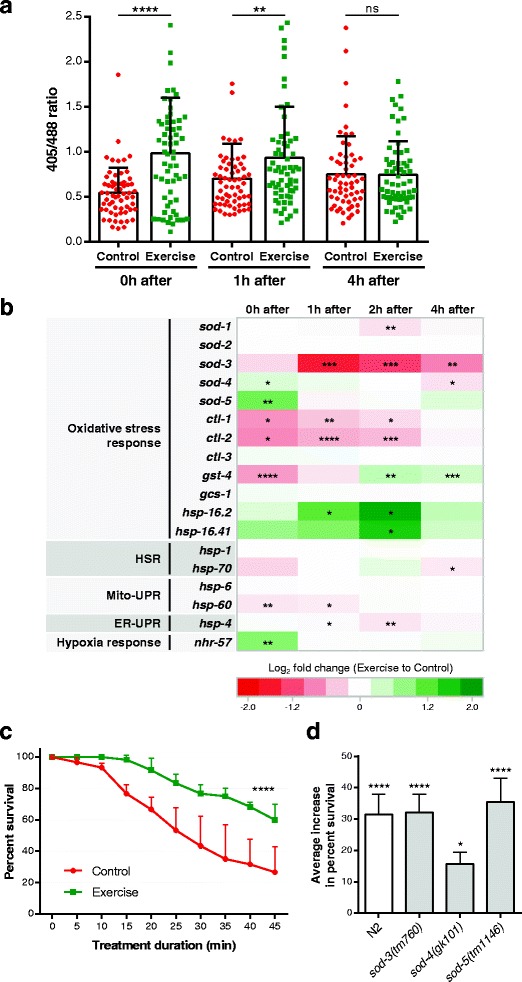
*C. elegans* swim exercise increases muscle mitochondrial oxidation and induces a specific transcriptional oxidative stress response. **a** Relative mitochondrial oxidation level of *P*_*myo-3*_ *mito-roGFP*-expressing animals at different time points post-exercise (higher 405/488 ratios indicate increased oxidation levels). We took confocal images of body wall muscle at both 405 nm and 488 nm excitation and used the mean fluorescence intensities of mitochondrial regions (40–50 regions per animal) to calculate the 405/488 ratios. Each point represents the 405/488 ratio average from a single animal (*n* = 60–63 animals); statistical significance was determined by unpaired two-tailed Student’s *t* test. **b** Heat map summarizing quantitative polymerase chain reaction results in N2 animals for commonly used stress reporter genes at different time points post-exercise (*n* = 5 independent trials). Expression data are presented as the log_2_ fold change of exercise samples relative to control samples in a color gradient from *red* (downregulation) to *dark green* (upregulation). *White* represents no change in expression levels. Paired two-tailed Student’s *t* tests were used to compare relative expressions of control versus exercise samples at each time point. See Additional file 1 and Additional file 4A–F for detailed results for each gene. **c** Percentage of surviving N2 animals during treatment with 3 mM juglone 4 hours post-exercise (*n* = 60 animals). **d** Average increase in percent survival during treatment with 3 mM juglone 4 hours post-exercise of N2, *sod-3(tm760)*, *sod-4(gk101)*, and *sod-5(tm1146)* exercised animals relative to control counterparts (*n* = 60 animals). The average increase in percent survival was calculated from all the time points between 15 and 45 min of treatment duration. Note that the reduced survival benefit of exercised *sod-4* mutants was not due to a reduced swimming capacity relative to N2 and the other *sod* mutants. See Additional file 3B–D for detailed survival curves of *sod* mutants. Statistical significance in (c) and (d) determined by a log-rank test comparing control versus exercise survival curves. *P < 0.05; **P < 0.01; ***P < 0.001; ****P < 0.0001. *ER-UPR* endoplasmic reticulum unfolded protein response, *HSR* heat shock response, *ns*, non-significant, *mito*-*UPR* mitochondrial unfolded protein response

It is well known that physical exercise is associated with oxidative stress in human and mammalian muscle [20, 21]. To address whether the generation of reactive oxygen species (ROS) and an oxidative stress response in muscle are features of *C. elegans* swim exercise, we first took advantage of the reduction-oxidation-sensitive green fluorescent protein (roGFP), which allows for ratiometric quantification of redox changes in live cells [22]. Using a *C. elegans* strain that expresses roGFP specifically in the body wall muscle mitochondria [23], we determined that immediately after the swim exercise, the muscle mitochondrial matrix of swimmers was significantly more oxidized than the mitochondrial matrix in matched non-exercise controls (Fig. 3a). The increased oxidation level was maintained 1 hour after the end of exercise, but by 4 hours post-exercise the mitochondrial oxidative environment again matched control levels (Fig. 3a). Thus, as is true in mammals, acute physical exercise in *C. elegans* increases mitochondrial oxidation in muscle, and nematodes are able to adapt/clear elevated oxidation levels within a few hours to re-establish the mitochondrial oxidative environment to basal levels.

### Changes in specific oxidative stress response transcripts accompany swim exercise

Having documented evidence of a transient oxidative increase in *C. elegans* body wall muscle mitochondria after acute exercise, we used quantitative polymerase chain reaction (qPCR) to quantitate transcript levels of selected cellular oxidative stress-responsive genes immediately after the swim and during recovery. We first focused on key antioxidant defense genes encoding superoxide dismutase (SOD, the sole detoxifying enzyme for superoxide) and catalase (detoxifies H_2_O_2_). The *C. elegans* genome encodes five SODs: SOD-1 (constitutive) and SOD-5 (inducible) are cytoplasmic, SOD-2 (constitutive) and SOD-3 (inducible) are mitochondrial, and SOD-4 is extracellular [24]. There are three *C. elegans* catalases, CTL-1 (cytoplasmic), CTL-2 (peroxisomal), and CTL-3 [25, 26]. Our quantitation revealed that *sod-4* and *sod-5* are significantly upregulated immediately after the swim exercise, but rapidly return to control levels in later time points; *sod-3*, *ctl-1*, and *ctl-2* are significantly downregulated following exercise; and *sod-1*, *sod-2*, and *ctl-3* transcripts showed little or no change between control and exercised animals (Fig. 3b and Additional file 1A–H). We also analyzed the expression of *gst-4* and *gcs-1*, which encode phase II detoxification enzymes involved in glutathione antioxidant defense [27–29]. While the transcript levels of *gst-4* (mainly expressed in body wall muscle [30]) changed significantly during the swim/recovery period, the levels of *gcs-1* (mainly expressed in the pharynx and intestine [29]) did not (Fig. 3b; Additional file 1I, J). Finally, we also included the heat shock protein genes *hsp-16.2* and *hsp-16.41* (genes divergently transcribed from a shared promoter [31]) in our qPCR analysis, given their previous documentation as oxidative stress-responsive reporters in *C. elegans* (in addition to heat shock inducibility) [32–34]. Both *hsp-16.2* and *hsp-16.41* showed a marked upregulation in swim-exercised animals, with peak expression occurring at 2 hours post-exercise (Fig. 3b; Additional file 1K, L).

Importantly, the expression changes we documented were exercise-dependent rather than attributable to liquid exposure, given that paralyzed *unc-54* mutants placed in M9 buffer for 90 min did not show similar expression patterns for the most strongly affected transcripts in wild-type animals (Additional file 2). Our sampling of oxidative stress response machinery thus shows that an acute swim exercise in *C. elegans* induces a “mixed” oxidative stress transcriptional response in the hours post-exercise, featuring upregulation of some oxidative stress genes (e.g., *sod-4*, *sod-5*, *gst-4*, *hsp-16.2*, *hsp-16.41*) and concomitant downregulation of others (e.g., *sod-3*, *ctl-1*, *ctl-2*).

Mild stresses are thought to induce protective defenses via a process called hormesis, and we wondered if a swim session and its associated physiological changes might enhance robustness. To address whether acute swim bouts might enhance overall oxidative defenses in exercised animals, we treated nematodes at 4 hours post-exercise with juglone, a xenobiotic compound that generates high levels of ROS [35]. We found that a significantly higher proportion of swim-exercised animals survived the juglone treatment as compared to their non-exercised control counterparts (Fig. 3c). This exercise benefit was mostly gone by 24 hours post-exercise, even though exercised animals still exhibited a trend for increased juglone resistance (Additional file 3A).

Given the crucial role of SODs in addressing high levels of superoxide, we investigated whether the SOD genes modulated by swim exercise in *C. elegans* (i.e., *sod-3*, *sod-4*, and *sod-5*) had any role in the increased survival to juglone treatment. We found that exercised *sod-3* and *sod-5* mutants exhibited increased survival relative to their control counterparts, identical to N2 animals (30–35%). However, exercised *sod-4* mutants exhibited a diminished response of only a 15% survival increase (Fig. 3d; Additional file 3B–D). We conclude that even a single swim exercise bout is associated with physiological changes, partially dependent on the extracellular SOD-4, that have a role in protection against a subsequent lethal oxidative stress.

### Swim exercise does not induce a generalized stress response in *C. elegans*

To address how generally stress responses might be activated during the swim, we determined expression levels by qPCR of commonly studied reporter genes that are activated by multiple types of stress: *hsp-1* and *hsp-70* for heat shock response (HSR) [36, 37]; *hsp-6* and *hsp-60* for mitochondrial unfolded protein response (mito-UPR) [38]; *hsp-4* for endoplasmic reticulum unfolded protein response (ER-UPR) [39]; *nhr-57* for hypoxia response [40]; and *gpdh-1* and *nlp-29* for osmotic response [41, 42].

Our qPCR data suggest that swimming in M9 buffer for 90 min does not induce short-term HSR, mito-UPR, or ER-UPR because the reporter genes tested exhibited no induction in exercised animals (some of them were even slightly downregulated at specific time points) (Fig. 3b; Additional file 4A–E). Interestingly, we found that hypoxia-induced reporter *nhr-57* was significantly upregulated immediately after swim exercise and then returned to control levels in the following time points (Fig. 3b; Additional file 4F). Because *nhr-57* upregulation did not occur when *unc-54* mutants were exposed to M9 buffer (Additional file 4G), the expression changes can be attributed to exercise rather than to transfer into liquid. Data suggest that although hypoxia-associated signaling and responses occur during the swim, hypoxia signaling may be rapidly reversed upon return to a solid environment. Regarding osmotic stress reporter genes, both *nlp-29* and *gpdh-1* transcripts exhibited a highly dynamic expression pattern even in non-exercised control animals (Additional file 4H, I), suggesting that under our experimental setup, physical manipulations, rather than osmotic stress, drove their expression.

Overall, our data establish that a 90 min swim in M9 buffer does not lead to full-blown transcriptional activation of multiple stress pathways. Rather, specific and distributed exercise-dependent transcriptional changes accompany the acute swim bout. Moreover, the exercise experience is associated with enhanced oxidative stress defenses, suggesting that specific modulation of oxidative stress pathways may suffice to provide significant physiological benefit at least during the hours following the exercise bout.

### Transcriptional changes are consistent with reduced glucose metabolism after swim exercise

**Fig. 4:**
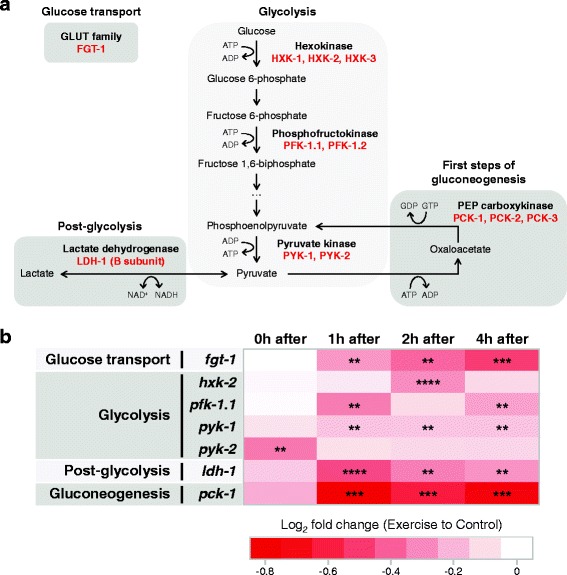
Transcriptional changes in *C. elegans* are consistent with reduced glucose metabolism after swim exercise. **a** Diagram of glucose metabolism highlighting the steps that we chose for our expression analysis. *C. elegans* proteins are shown in *red*. For simplicity, several metabolic steps are omitted. **b** Heat map summarizing quantitative polymerase chain reaction results in N2 animals at different time points post-exercise for glucose metabolic genes (*n* = 5 independent trials). Expression data is presented as log_2_ fold change of exercise samples relative to control samples in a color gradient from *red* (downregulation) to *white* (no change). Paired two-tailed Student’s *t* tests were used to compare relative expressions of control versus exercise samples at each time point. See Additional file 5 for detailed results for each gene. **P < 0.01; ***P < 0.001; ****P < 0.0001

The two main sources of energy during physical exercise are carbohydrates and fats. Therefore, we analyzed how acute swim exercise might affect gene expression of key members of both *C. elegans* carbohydrate and fat metabolism. To evaluate potential carbohydrate utilization, we focused our analysis on glucose metabolism: glucose transport, glycolysis, and gluconeogenesis (Fig. 4a). We found persistent downregulation of transcript levels of the main *C. elegans* glucose transporter, encoded by *fgt-1*, between 1 and 4 hours post-exercise (Fig. 4b; Additional file 5A). Expression of three essential glycolytic enzymes was also significantly downregulated in exercised animals at different time points: *hxk-2*, hexokinase (first enzyme of glycolysis); *pfk-1.1*, phosphofructokinase (rate-limiting enzyme of glycolysis); and *pyk-1* and *pyk-2*, pyruvate kinases (last enzymes of glycolysis) (Fig. 4b; Additional file 5B–E). *ldh-1*, encoding the B subunit of lactate dehydrogenase, which catalyzes the interconversion of pyruvate and lactate in a post-glycolysis process, was also downregulated between 1 and 4 hours after swim exercise (Fig. 4b; Additional file 5F). Finally, a similar temporal pattern of downregulation was observed for *pck-1*, a phosphoenolpyruvate carboxykinase that is the rate-limiting enzyme of gluconeogenesis (Fig. 4b; Additional file 5G; magnitude of downregulation greater for *pck-1*). The shared theme of expression downregulation of seven out of seven selected genes indicates that glucose metabolism as a whole may be reduced in the hours following acute swim exercise.

### Transcriptional changes suggest increased fat metabolism during exercise

**Fig. 5:**
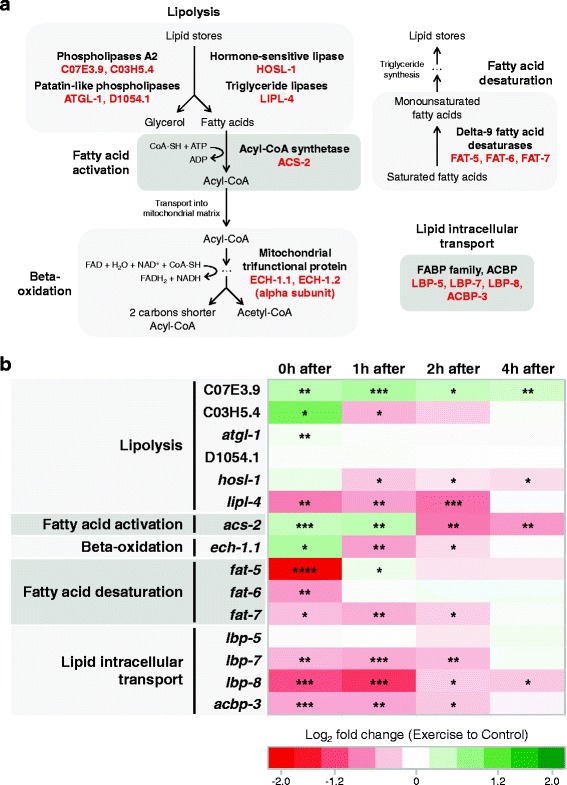
Transcriptional changes in *C. elegans* suggest increased fat metabolism during swim exercise. **a** Diagram of fat metabolism highlighting the steps that we chose for our expression analysis. *C. elegans* proteins are shown in *red*. For simplicity, several metabolic steps are omitted. **b** Heat map summarizing quantitative polymerase chain reaction results in N2 animals at different time points post-exercise for fat metabolic genes (*n* = 5 independent trials). Expression data is presented as log_2_ fold change of exercise samples relative to control samples in a color gradient from *red* (downregulation) to *dark green* (upregulation). *White* represents no change in expression levels. Paired two-tailed Student’s *t* tests were used to compare relative expressions of control versus exercise samples at each time point. See Additional file 6 for detailed results for each gene. *P < 0.05; **P < 0.01; ***P < 0.001; ****P < 0.0001

Fat metabolism is a complex network of enzymatic reactions that can lead to outcomes as diverse as lipid storage, fatty acid breakdown, lipid incorporation into cell membranes, or cell signaling, depending on the metabolic state and requirements of the organism. A large proportion of lipids in *C. elegans*, such as triglycerides, are stored in lipid droplets across different tissues. For fatty acids to be used as a source of energy, lipases have to break down triglycerides into glycerol and fatty acids. Fatty acids are then available for activation (conversion into acyl-coenzyme A(CoA)) followed by beta-oxidation, which generates acetyl-CoA that can enter the tricarboxylic acid cycle. We focused our analysis of lipid metabolism gene expression on fatty acid breakdown and lipid storage (Fig. 5a).

Our qPCR analyses revealed that transcripts of three *C. elegans* lipases, C07E3.9, C03H5.4, and *atgl-1* (the latter of which is documented to be expressed in muscle [43]), were significantly upregulated immediately after swim exercise. C07E3.9 maintained a higher expression level in exercised animals even after 4 hours, whereas C03H5.4 and *atgl-1* returned to control levels quickly after exercise completion. Other *C. elegans* lipases, known to be primarily expressed outside of muscle [43, 44], either did not change their expression (D1054.1) or were downregulated (*hosl-1* and *lipl-4*) after exercise (Fig. 5b; Additional file 6A–F). The enzyme responsible for fatty acid activation (acyl-CoA synthetase), encoded by *acs-2*, and the alpha subunit of the mitochondrial trifunctional protein responsible for the last three steps of fatty acid beta-oxidation, encoded by *ech-1.1*, were also upregulated at the end of swim exercise, followed by a post-exercise downregulation (Fig. 5b; Additional file 6G, H). Among the tested genes involved in fatty acid breakdown, an expression pattern emerged in which upregulation was observed specifically at the time point immediately after swim exercise. The transcriptional changes we quantitated suggest that *C. elegans* adapt gene expression in a manner expected to increase fat metabolism during physical exercise.

Consistent with the hypothesis of increased lipid breakdown during swim exercise, we also observed a strong downregulation of *C. elegans* delta-9 fatty acid desaturases, encoded by *fat-5*, *fat-6*, and *fat-7*, at the 0 hour time point (Fig. 5b; Additional file 6I–K). Downregulation of these desaturases as occurs during swim exercise should promote fat breakdown instead of fat synthesis/storage [45, 46]. Finally, we analyzed the transcriptional levels of genes encoding proteins involved in intracellular transport of lipids. Interestingly, two out of three fatty acid binding proteins, *lbp-7* and *lbp-8*, and one acyl-CoA binding protein, *acbp-3*, showed strong downregulation between 0 and 2 hours post-exercise (Fig. 5b; Additional file 6L–O). It has been proposed that LBP-7, LBP-8, and ACBP-3 can inhibit beta-oxidation by sequestering fatty acids [47, 48]. Furthermore, *acbp-3* mutants have been shown to contain lower levels of triglycerides accompanied by an increase in fatty acid beta-oxidation [49], suggesting that post-exercise downregulation of lipid binding proteins may promote fat breakdown.

### Lipid storage is depleted specifically in *C. elegans* body wall muscle after swim exercise

**Fig. 6:**
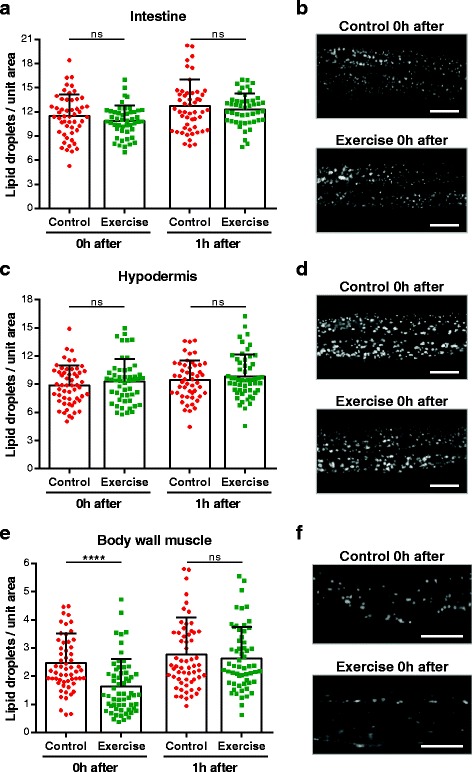
Lipid stores are depleted specifically in the body wall muscle of *C. elegans* after swim exercise. Quantification of the number of lipid droplets and representative confocal images at different time points post-exercise in tissue-specific Perilipin 1-GFP strains: intestine, *P*_*daf-22*_ *PLIN1::GFP* (**a**, **b**); hypodermis, *P*_*Y37A1B.5*_ *PLIN1::GFP* (**c**, **d**); and body wall muscle, *P*_*unc-54*_ *PLIN1::GFP* (**e**, **f**). Each point represents a single animal (*n* = 47–61 animals); statistical significance determined by unpaired two-tailed Student’s *t* test. Scale bars: 20 μm. ****P < 0.0001. *ns* non-significant

Our expression analyses strongly suggest that lipid catabolism increases in *C. elegans* during swim exercise, which led us to explore whether lipid stores in different *C. elegans* tissues are affected by physical exercise. To evaluate tissue-specific fat use, we used *C. elegans* strains in which lipid droplets were fluorescently labeled by Perilipin 1-GFP in a tissue-specific manner [50]. We measured lipid droplets in these strains via quantitative image analysis immediately after the swim, comparing exercised to non-exercise animals. While the number of lipid droplets in the intestine (Fig. 6a, b) or hypodermis (Fig. 6c, d) was not changed during exercise, we measured a significant decrease in the lipid droplets of body wall muscle immediately after swim exercise (Fig. 6e, f). Muscle lipid droplet number returned to control levels just 1 hour later (Fig. 6e), revealing the highly dynamic regulation of fat metabolism in exercised muscle. These results confirm that *C. elegans* increase fat breakdown during swim exercise in a tissue-specific manner, with intramuscular lipid reserves being used to fuel the high-energy swimming locomotion. Moreover, our data underscore that even a single swim bout suffices to induce physiological change in exercised muscle.

## Discussion

The development of invertebrate genetic exercise models holds great potential for enhancing understanding of the molecular mechanisms that mediate the system-wide health benefits of physical exercise. Here, we show that key features of acute mammalian exercise are conserved in *C. elegans*: increased muscular metabolic rate; locomotory fatigue after exercise; and increased muscle mitochondrial oxidation followed by transcriptional responses impacting particular oxidative stress defense genes, carbohydrate utilization genes, and fat metabolism genes. Consistent with the hypothesis that swim exercise has direct physiological consequences in *C. elegans*, we have directly visualized considerable depletion of muscle fat stores after a 90 min swim. Additionally, we find that a single exercise session can improve organism resistance to a toxic oxidative stress challenge, a striking demonstration of how even limited activity enhancement can improve organism robustness.

### Setting the experimental foundation of a *C. elegans* exercise model

Although multiple aspects of crawling versus swimming locomotion in *C. elegans* have been studied [13–15, 51], we provide here the first direct evidence for distinct energy costs. Using microcalorimetry we found that the difference in energy expenditure between activity and rest was higher for swimming than for crawling. We speculate that the lower oxygen availability in an aqueous solution, such as M9 buffer, compared to air (around 30 times lower by Henry’s law [52]) contributes to the differences observed in SMR between liquid and solid environments. In fact, when exposed to a 30-fold lower oxygen concentration, the *C. elegans* metabolic rate has been shown to reduce by about 2.5-fold [53], which matches the SMR difference observed in our results. Despite the lower oxygen availability in M9 buffer, our data also show that liquid exposure by itself for 90 min is not sufficient to induce a detectable hypoxia transcriptional response and that nematodes in liquid are able to significantly increase their metabolic rate when swimming starts.

The increase in energy expenditure during physical exercise is well documented to induce fatigue and exhaustion in humans [54]. *C. elegans* exhibited reduced crawling ability after a long swim but were able to quickly recover (within 1 hour) to normal movement levels. The behavioral change documented here after a single swim session, together with decreased body wall muscle fat stores, suggests a muscular fatigue phenotype due to depletion of energy stocks. Consistent with this idea, the intra-muscular lipid reserves in exercised animals returned to control levels in the same time frame as they resumed normal crawling speeds.

Another facet of a *C. elegans* swim is the increased oxidation in muscle mitochondria. The mitochondrial oxidation level in *C. elegans* body wall muscle increased after acute swim exercise, and needed 4 hours to return to basal levels. This recovery as reported by mito-roGFP does not have to involve mitochondrial turnover because the redox-dependent photo-shift of mito-roGFP is reversible [22], although increased mitophagy might be anticipated as a consequence of exercise [55, 56]. Our data establish that swimming is accompanied by a transient increase in oxidative conditions within mitochondria, similar to what occurs in exercised mouse and human muscle [21]. Possibly, mitochondrial ROS-induced signals might drive nuclear transcriptional changes for ROS-associated genes.

Interestingly, our results reveal upregulation of the hypoxia inducible factor 1 (HIF-1) target gene *nhr-57* during the swim, but not in paralyzed animals that are placed into liquid. Hypoxia reporter *nhr-57* is downregulated rapidly after swim cessation, suggesting the swim-associated hypoxia signaling may apply only during exercise. Multiple components of the HIF-1 pathway are upregulated in human skeletal muscle after acute exercise [57–60] and a similar response may occur in *C. elegans*, with swim exercise creating a localized hypoxic environment in the body wall muscle due to the increase in metabolic rate and oxygen consumption.

### Swim exercise induces a specific oxidative transcriptional response

We examined levels of transcripts for all *C. elegans* superoxide-detoxifying SOD genes before, immediately after, and up to 4 hours post-exercise to generate a snapshot of how transcription for the full superoxide defense system changes with acute exercise and to address whether ROS at specific cellular locations might be differentially implicated in intracellular responses. Our findings revealed exercise-associated differences in the transcription of SOD genes that encode compartment-specific defenses: extracellular *sod-4* and inducible cytoplasmic *sod-5* were transiently upregulated, but mitochondrial *sod-2* transcripts did not change, and inducible mitochondrial *sod-3* levels decreased, despite the documented elevated oxidation that occurs in the muscle mitochondria. Although qPCR measures must be interpreted in light of the fact that these data represent whole-animal mRNA levels (such that tissue-specific changes might be masked), and transcript levels do not directly indicate protein activity, our observations reflect physiological responses that suggest that muscle superoxide production is not sufficient to induce an immediate increase in the transcription of mitochondrial SOD genes. Interestingly, although mitochondria are generally considered to be major ROS production sites, mammalian mitochondria have actually been measured to generate relatively little superoxide during acute exercise [61, 62]. That inducible mitochondrial *sod-3* transcripts were reduced after the swim raises the possibility that the muscle might act to increase mitochondrial superoxide signaling, which is known to help promote exercise adaptation [63].

In addition to reproducible elevation of *sod-4* and *sod-5* transcripts, we found that the oxidative stress reporters *gst-4* and *hsp-16.2/41* were also upregulated consequent to single swim bouts, with differences in the timing and magnitude of upregulation among these genes. Future protein level changes with tests of biochemical activities will be needed to determine the actual cellular changes in compartment-specific antioxidant defense. Nonetheless, our data show that swimming in *C. elegans* is associated with both increased oxidation in muscle mitochondria and modulation of multiple antioxidant defense nuclear transcripts, supporting the fact that ROS constitute a significant component of in vivo swim responses.

Over the short term, there were no transcriptional changes for the tested mito-UPR, ER-UPR, and heat shock chaperones (apart from those also known to be induced by oxidative stress); some osmotic stress reporter changes (*gpdh-1* and *nlp-29)* occurred after the swim, but with temporal similarity to those in control animals, suggesting that these transcriptional fluctuations might reflect the mechanical stress associated with plate transfers. A main point is that transcriptional elevation across multiple major stress pathways is not a feature of an acute swim. Rather, our findings indicate that specific and reproducible transcriptional responses are activated during the acute swim phase and immediately after.

### Metabolic restructuring may be signaled via an acute exercise bout

Our qPCR studies suggest that lipolysis and fatty acid beta-oxidation are upregulated during swim exercise, while glycolysis may be diminished or unchanged, indicating that the preferred source of energy for *C. elegans* during swim exercise is fat. Interestingly, using GFP-tagged perilipin reporters, we documented an increase in lipid breakdown that occurred specifically in the body wall muscle, with intestinal and hypodermal lipid reserves not being affected by a single exercise bout. Although the intestine and hypodermis are the main tissues for lipid storage in *C. elegans* [64, 65], we detected significant transcriptional changes in fat metabolic genes that likely occurred in the body wall muscle. Thus, given whole-animal transcript measures, muscle-specific expression changes in lipid metabolism genes after exercise might be even more accentuated than we were able to detect. We note that in humans, intramuscular triglyceride stores are reduced by approximately 60% after acute exercise [66, 67], highlighting another feature of acute exercise that is similar between *C. elegans* and humans.

Our qPCR analyses of metabolic genes revealed that, in most cases, genes with similar functions or involved in the same pathway responded to swim exercise in a similar fashion – there was elevated expression of fat catabolic genes and reduced expression of fat anabolic genes immediately after the 90 min swim. Moreover, most fat metabolic genes showed transcriptional modulation immediately post-exercise (and likely during exercise) but then returned to control levels within 4 hours, suggesting a quick response of fat metabolism to high energy demands.

By contrast, glucose metabolism seemed largely unchanged immediately after exercise, but a theme of transcriptional downregulation became apparent in the following hours (1–4 hours). This post-exercise pattern suggests that acute exercise in *C. elegans* might induce medium- to long-term changes in glucose metabolism. In mammalian exercise systems, carbohydrate sources have been shown to be preserved during the recovery period [68]. Interestingly, downregulation of carbohydrate metabolism has also been reported in *Drosophila* following endurance exercise [6], pointing to a conserved exercise-dependent metabolic response in invertebrate and vertebrate models. Glucose and lipid contributions to the energy expended during physical exercise vary widely depending on the exercise duration and intensity. Lipid oxidation in mammals is maximized in prolonged, moderate intensity exercise [68], implying that a 90 min swim in *C. elegans* may fit into the category of extended moderate intensity exercise (possibly an endurance-type activity).

### Just one swim is good for a worm

We showed that a single *C. elegans* swim bout has a protective role against a juglone-induced oxidative stress challenge delivered 4 hours after activity cessation. Somewhat remarkably, acute exercise in *C. elegans* may activate a sufficient hormetic response to confer beneficial effects. We speculate that this resistance might be achieved from the mild oxidative stress response generated by swimming. In fact, we show that this exercise-induced survival is partially dependent on the *C. elegans* extracellular SOD-4, which is of particular interest given previous mouse studies reporting a specific increase in extracellular SOD after a single bout of exercise [69] and a role for extracellular SOD in systemic exercise benefits [70, 71]. Our findings that oxidative stress is transiently increased and that there is a specific network of antioxidant gene expression changes support the contention that physiological ROS signaling can have powerful positive consequences [72, 73].

### A straightforward exercise model, with short-term activity-based changes in common with fly and mammalian training

*C. elegans* swim behavior has been studied in detail at the genetic and behavioral levels [13–15, 19, 51] and swimming capacity has been used as a mobility endurance test for *C. elegans* [74], but the molecular outcomes of short swims have not been previously investigated. Chuang et al. [75] described an exercise paradigm for *C. elegans* based on simultaneous swimming and exposure to an electrical field for 10 min per day. The electro-swim model, however, includes the caveat that the effects of the electrical field exposure may be difficult to experimentally separate from the effect of movement per se, which might complicate the elaboration of physiological factors critical to exercise health benefits. We suggest that simple swim regimens, which can be monitored for continuous activity over defined times using available computational programs if needed [76], will be a productive approach toward dissecting physiologically relevant changes and inter-tissue signaling that promotes vigor and extends healthspan. The documentation of adaptation outcomes resulting from chronic exercise will require extended measurement of metabolic and endurance changes that lie beyond the focus of this paper, but constitute the logical extension of the single swim changes we report here.

## Conclusions

We show in this study that *C. elegans* is a suitable model for exercise research due to the conservation of key mammalian exercise features in response to a single swim session. The establishment of a characterized exercise protocol opens the exciting possibility of exploring the molecular basis of system-wide benefits of physical exercise in an invertebrate model with a short lifespan. Thus, life-long effects of exercise can be measured at a cellular, tissue, and organismal level in unprecedented ways.

## Methods

### *C. elegans* strains and maintenance

The *C. elegans* strains used in this study were: N2, CB1092 *unc-54(e1092) I*, KWN122 *pha-1(e2123ts) III; him-5(e1490) V; rnyEx061[pDJ7(P*_*myo-3*_ *mito-roGFP) pCL1(pha-1+)]*, ZB4581 *sod-3(tm760) X*, ZB4563 *sod-4(gk101) III*, ZB4587 *sod-5(tm1146) II*, XD3971 *xdIs143[P*_*daf-22*_ *PLIN1::GFP rol-6(su1006)]*, XD2458 *xdIs56[P*_*Y37A1B.5*_ *PLIN1::GFP rol-6(su1006)]*, and XD1875 *xdIs26[P*_*unc-54*_ *PLIN1::GFP rol-6(su1006)]*. ZB4581 was generated by outcrossing MQ1476 *sod-2(ok1030) I; sod-3(tm760) X* to N2 six times and PCR selection of single *sod-3* mutants. ZB4563 and ZB4587 were generated by outcrossing GA416 *sod-4(gk101) III* and GA503 *sod-5(tm1146) II*, respectively, to N2 five times. Most animals from XD3971, XD2458, and XD1875 strains displayed only a weak roller phenotype and thus were able to crawl and swim fairly normally. We maintained nematodes at 20 °C on NGM agar plates seeded with live *Escherichia coli* OP50-1 (streptomycin-resistant strain) as previously described [77], unless otherwise stated.

### Microcalorimetry

We performed microcalorimetry assays as previously described [17] with the following modifications. Given the high number of animals needed for each measurement, we grew *C. elegans* on nutrient agar plates seeded with live *E. coli* K12, which sustain higher density populations than strain OP50. We bleached young adult populations to harvest eggs, and then added these to unseeded NGM agar plates for overnight hatching at 20 °C. The next day, we washed off synchronized L1 larvae and transferred them to NGM agar plates seeded with live *E. coli* OP50 (this was considered experimental day 0). On day 2, we added 5-fluorodeoxyuridine (Sigma-Aldrich, St. Louis, MO, USA) to the plates to a final concentration of 100 μM to prevent progeny development. On day 4, we washed young adult *C. elegans* off the plates, followed by three more M9 buffer washes to remove most bacteria (note that it is critical that bacteria are not present in these studies, as their metabolism would confound data interpretation). We used specific volumes of nematodes for microcalorimetry measurements (1000–1500 animals per sample) in a Thermal Activity Monitor 2277 (Thermometric AB, Jarfalla, Sweden), which accommodates four measuring units. Each unit received a reference ampoule (without animals) and a test ampoule (with animals). We sunk the measuring units into a precisely temperature-regulated water bath at 20 °C and monitored heat flows, obtaining stable measurements after 1 hour of thermal equilibration. We used the average heat output (in μW) between 1 and 2 hours for all our quantifications, normalizing heat output values to the total protein content (in mg) of each sample, calculated using the Pierce BCA Protein Assay Kit (Thermo Fisher Scientific, Waltham, MA, USA). Note that due to experimental constraints (sample preparation and microcalorimeter thermal equilibrations), the AMR of swimming animals included measurements after the 90 min time point, and therefore some animals might have already entered the episodic phase of active swimming alternating with periods of quiescence [19]. This could underestimate energy use for the 90 min continuous swim.

We conducted microcalorimetry measurements for both solid and liquid environments. For the solid environment, we covered the entire inside of the 20 mL ampoule with a thin layer of NGM agar and used small pieces of filter paper to absorb the extra M9 buffer transferred with the nematodes. For the liquid environment, we added animals to M9 buffer in the ampoule. For SMR calculation, we treated wild-type N2 *C. elegans* with 10 mM levamisole (Sigma-Aldrich, St. Louis, MO, USA) for 5 min before they were added to ampoules that had 10 mM levamisole included either in the NGM agar or the M9 buffer.

### Acute exercise protocol

We maintained *C. elegans* carefully at 20 °C for several generations without being starved prior to the exercise protocol. We synchronized the population by allowing gravid hermaphrodites to lay eggs in a seeded NGM agar plate for 3 hours before removing the parents. In the case of *unc-54* mutants, we synchronized by bleaching a population of young adults and added harvested eggs to a seeded NGM agar plate. In both cases, we considered the day of egg deposition to be experimental day 0. On day 3, we moved animals to new seeded plates (25–30 animals per plate). On day 4, we used all *C. elegans* in a single plate for a single experimental time point: for exercise, we transferred animals to M9 buffer covering an unseeded NGM agar plate for 90 min; for the non-exercise control, we transferred animals to an unseeded NGM agar plate for 90 min. We returned both exercise and control animals to seeded NGM agar plates after the 90 min.

### Crawling assay

We exercised wild-type N2 nematodes for 5, 30, 60, or 90 min. We then recorded 3 min videos at different time points post-exercise as animals crawled on seeded NGM agar plates (four animals per plate) with a Prosilica GC 1380 camera (Allied Vision, Burnaby, BC, Canada) connected to a Macro Zoom 7000 lens (Navitar, Rochester, NY, USA) using the StreamPix 5 software (NorPix, Montreal, QC, Canada). We calculated recovery crawl distance by manually tracking worm movement in ImageJ, and then calculated the recovery crawl distance ratio by dividing the average distance of exercised animals by the average distance of non-exercised paired controls.

### Confocal microscopy

After the acute swim exercise on day 4, we fixed *C. elegans* at different time points in 2% paraformaldehyde/phosphate buffered saline for 30 min at room temperature and then stored them at 4 °C in M9 buffer until imaging. We performed confocal imaging with a CSU-X1 spinning disk unit (Yokogawa, Sugar Land, TX, USA) mounted to an Axio Imager Z1 microscope (Zeiss, Oberkochen, Germany) using MetaMorph Premier software (Molecular Devices, Sunnyvale, CA, USA).

For *P*_*myo-3*_ *mito-roGFP* transgenic animals (KWN122 strain), we imaged body wall muscle cells at both 405 nm and 488 nm excitation with a 525/50 nm emission filter. We performed image analysis in ImageJ by manual selection of 40–50 mitochondrial regions per image, quantitating the mean fluorescence intensities of the exact same mitochondrial regions in both 405 nm and 488 nm images, and calculating the 405/488 ratio for each region. For each animal we generated a single 405/488 ratio value, which corresponded to the average of all 40–50 of the 405/488 ratios.

For tissue-specific *PLIN1::GFP* transgenic animals (XD3971, XD2458, and XD1875 strains), we imaged intestine, hypodermis, or body wall muscle at 488 nm excitation with a 525/50 nm emission filter. We quantitated the number of lipid droplets manually in ImageJ and normalized to the area analyzed in each image.

### RNA extraction and quantitative PCR

We collected N2 *C. elegans* into TRIzol Reagent (Thermo Fisher Scientific, Waltham, MA, USA) at different time points before or post-exercise (25–30 animals per sample) and immediately froze animals in liquid nitrogen. We stored samples at −80 °C until further processing. After three freeze-thaw cycles with liquid nitrogen/37 °C heat block, we extracted total RNA following the manufacturer’s instructions (Thermo Fisher Scientific). We synthesized cDNA from 400 ng of total RNA using the SuperScript III First-Strand Synthesis System (Thermo Fisher Scientific, Waltham, MA, USA).

We carried out quantitative PCR using diluted cDNA, SYBR Select Master Mix for CFX (Thermo Fisher Scientific, Waltham, MA, USA), and 0.5 μM of gene-specific primers (Additional file 7) in a 7500 Fast Real-Time PCR System (Thermo Fisher Scientific, Waltham, MA, USA), calculating relative expression using the ΔΔCt method [78] with *cdc-42* and Y45F10D.4 as reference genes [79].

We generated heat maps in Microsoft Excel by conditional formatting of expression data, with the log_2_ fold change of exercise samples relative to control samples presented in a color gradient from red (downregulation) to dark green (upregulation). White represents no change in expression levels.

### Juglone treatment

We treated *C. elegans* 4 hours post-exercise with 3 mM juglone (Sigma-Aldrich, St. Louis, MO, USA) in M9 buffer. For N2 animals 24 hours post-exercise, we used 4 mM juglone in M9 buffer due to an increased resistance to juglone of older animals. For each trial, we exposed 20 exercise or control animals to juglone, and scored death at regular intervals. Animals were considered dead when they mounted no response to prodding.

### Statistical analysis

The data in this study are presented as the average ± standard deviation. We performed a minimum of three independent trials for each experiment. The specific number of data points used for each statistical analysis are presented in figure legends. Statistical significance was determined by two-tailed Student’s *t* test, except for survival curves upon juglone treatment, for which log-rank test was used. For qPCR results, only log_2_ fold changes ≤ −0.15 or ≥0.15 were tested for statistical significance.

### Note on *C. elegans*/mammalian muscle differences

There are some differences between invertebrate and mammalian muscle that should be noted. *C. elegans* body wall muscles are not multinucleated, extend out to neurons via muscle arms, have some molecular differences in muscle proteins, and show no distinction between slow and fast twitch fibers [7–9]. *C. elegans* muscle also lacks muscle satellite cells, so aging and repair studies are more analogous to mammalian situations in which stem cells are depleted. The small size of the animal means that it is not easily feasible to conduct some single animal measures – for example, oxygen consumption and microcalorimetry require population assays. Although electrophysiological assays are possible in *C. elegans* muscle and neurons, the tiny size of the animal makes this a challenge reserved for a few highly skilled laboratories. Physical tissue dissection is also a challenge.

## Additional files

Additional file 1: Changes in specific oxidative stress response transcripts accompany swim exercise in *C. elegans*. (**A**–**L**) qPCR results in N2 animals before and at different time points post-exercise for oxidative stress reporter genes (*n* = 5 independent trials). Note the different y-axis scales between figure panels (particularly K and L). *hsp-16.2* and *hsp-16.41* are documented to be induced under both oxidative stress and heat shock. We calculated relative expression by normalization to reference genes followed by normalization to the time point before exercise. We used paired two-tailed Student’s *t* tests to compare relative expression of control versus exercise samples at each time point. *P < 0.05; **P < 0.01; ***P < 0.001; ****P < 0.0001. (PDF 260 kb)

Additional file 2: Transcript quantitation in paralyzed *unc-54* mutants indicate that the oxidative stress response is exercise-dependent. (**A**–**D**) qPCR results in *unc-54* mutants before and at different time points after a 90 min exposure to M9 buffer for oxidative stress reporter genes (*n* = 3 independent trials). Note the different y-axis scales between figure panels; scales presented are the same as in Additional file 1 for the respective genes to allow for a direct comparison between N2 and *unc-54* mutants. We calculated relative expression by normalization to reference genes followed by normalization to the time point before exercise. We used paired two-tailed Student’s *t* tests to compare relative expression of control versus M9 buffer samples at each time point. *P < 0.05. (PDF 217 kb)

Additional file 3: Post-exercise increased survival to juglone treatment is partially dependent on *sod-4*. (**A**) Percentage of surviving N2 animals during treatment with 4 mM juglone 24 hours post-exercise (*n* = 60 animals). Exercised animals still exhibited a trend for increased survival 24 hours post-exercise, although this was statistically non-significant (*P* = 0.067). We used a higher concentration of juglone for this particular experiment given an increased resistance to juglone of older N2 animals. Percentage of surviving (**B**) *sod-3(tm760)*, (**C**) *sod-4(gk101)*, and (**D**) *sod-5(tm1146)* animals during treatment with 3 mM juglone 4 hours post-exercise (*n* = 60 animals). Statistical significance determined by log-rank test. *P < 0.05; ****P < 0.0001. *ns* non-significant. (PDF 233 kb)

Additional file 4: Swim exercise does not induce a generalized stress response in *C. elegans*. qPCR results before and at different time points post-exercise for (**A**, **B**) heat shock response, (**C**, **D**) mitochondrial unfolded protein response, (**E**) endoplasmic reticulum unfolded protein response, (**F**, **G**) hypoxia response, and (**H**, **I**) osmotic response reporter genes. All panels refer to N2 animals (*n* = 5 independent trials), except (G), which reports for *unc-54* mutants (*n* = 3 independent trials). Note the different y-axis scales between figure panels; scales presented in (F) and (G) are the same to allow for a direct comparison between N2 and *unc-54* mutants. We calculated relative expression by normalization to reference genes followed by normalization to the time point before exercise. We used paired two-tailed Student’s *t* tests to compare relative expression of control versus exercise samples at each time point. *P < 0.05; **P < 0.01. (PDF 241 kb)

Additional file 5: Expression changes are consistent with reduced glucose metabolism after swim exercise in *C. elegans*. (**A**–**G**) qPCR results in N2 animals before and at different time points post-exercise for glucose metabolic genes (*n* = 5 independent trials). Note the different y-axis scale in (E) compared to all other figure panels. We calculated relative expression by normalization to reference genes followed by normalization to the time point before exercise. We used paired two-tailed Student’s *t* tests to compare relative expression of control versus exercise samples at each time point. **P < 0.01; ***P < 0.001; ****P < 0.0001. (PDF 227 kb)

Additional file 6: Expression changes are consistent with increased fat metabolism during swim exercise in *C. elegans*. (**A**–**O**) qPCR results in N2 animals before and at different time points post-exercise for fat metabolic genes (*n* = 5 independent trials). Note the different y-axis scales between figure panels. We calculated relative expression by normalization to reference genes followed by normalization to the time point before exercise. We used paired two-tailed Student’s *t* tests to compare relative expression of control versus exercise samples at each time point. *P < 0.05; **P < 0.01; ***P < 0.001; ****P < 0.0001. (PDF 269 kb)

Additional file 7: Primers used for qPCR. (DOCX 16 kb)

Additional file 8: Raw data. (XLSX 124 kb)
