# Supplementary figures and images for: Single swim sessions in C. elegans induce key features of mammalian exercise
Source: BMC Biol. 2017 Apr 10;15:30. doi: 10.1186/s12915-017-0368-4 (PMC5385602; doi:10.1186/s12915-017-0368-4)

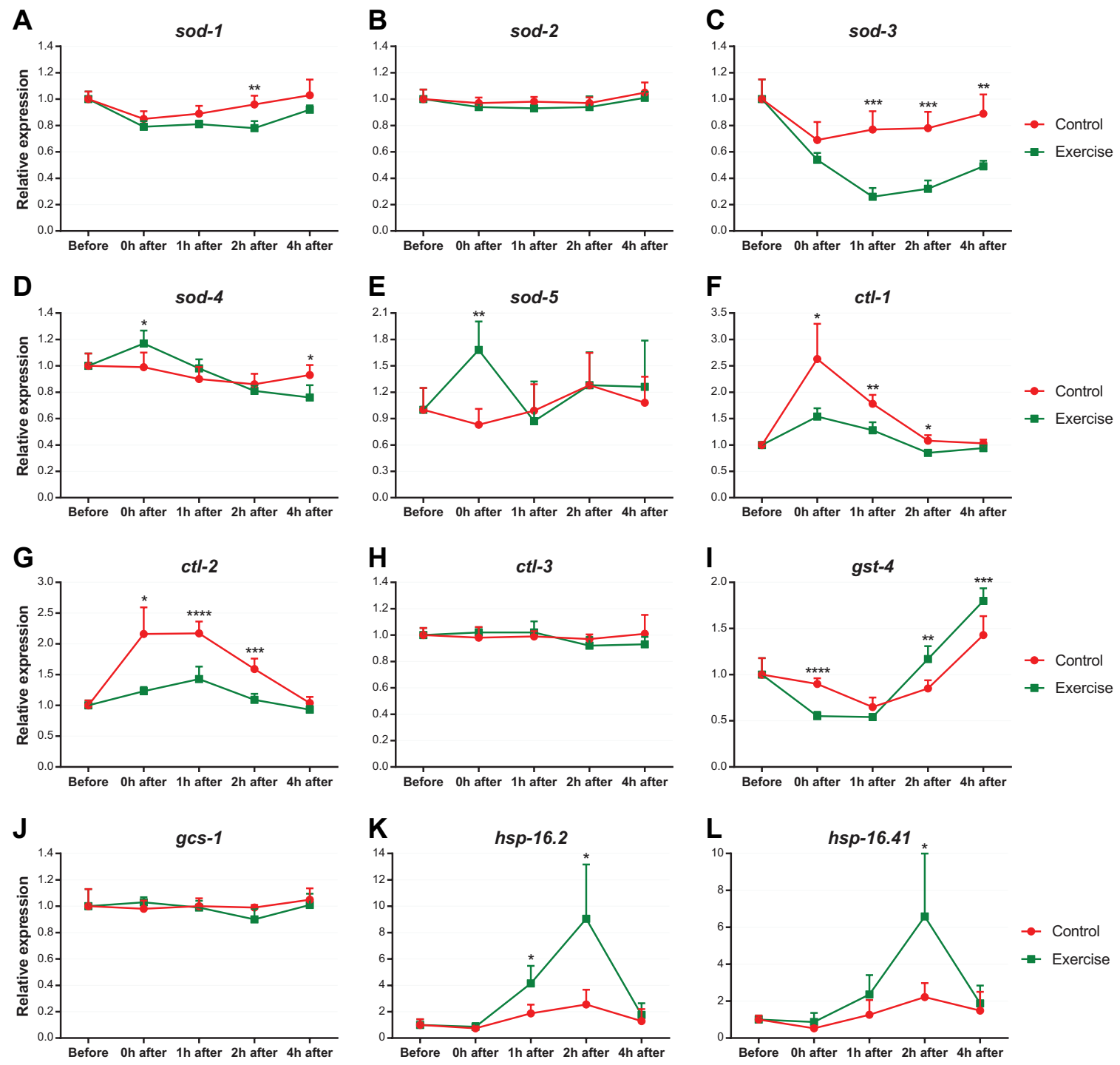

Supplement: Supplementary file 1 — Changes in specific oxidative stress response transcripts accompany swim exercise in C. elegans. (A–L) qPCR results in N2 animals before and at different time points post-exercise for oxidative stress reporter genes (n = 5 independent trials). Note the different y-axis scales between figure panels (particularly K and L). hsp-16.2 and hsp-16.41 are documented to be induced under both oxidative stress and heat shock. We calculated relative expression by normalization to reference genes followed by normalization to the time point before exercise. We used paired two-tailed Student’s t tests to compare relative expression of control versus exercise samples at each time point. *P < 0.05; **P < 0.01; ***P < 0.001; ****P < 0.0001. (PDF 260 kb) [file 12915_2017_368_MOESM1_ESM.pdf]

**A***hsp-16.2*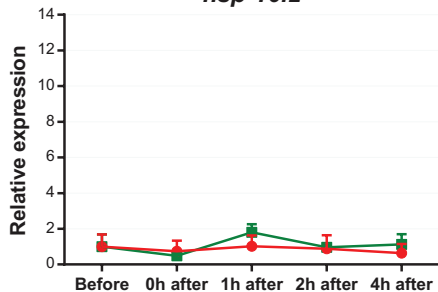**B***gst-4*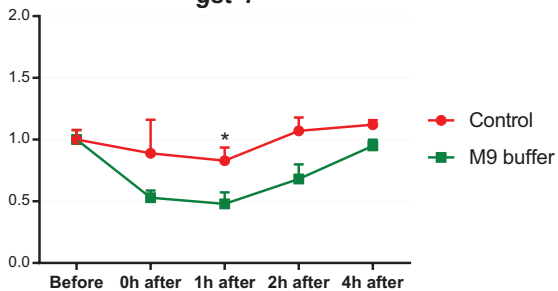**C***sod-3*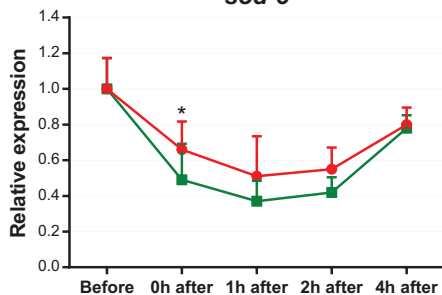**D***sod-5*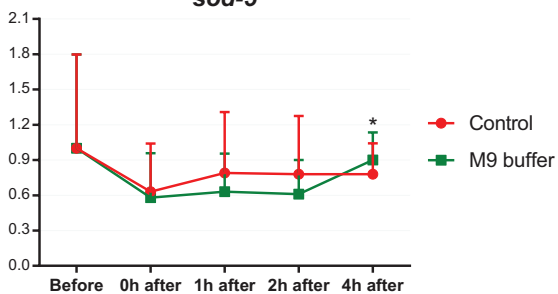

Supplement: Supplementary file 2 — Transcript quantitation in paralyzed unc-54 mutants indicate that the oxidative stress response is exercise-dependent. (A–D) qPCR results in unc-54 mutants before and at different time points after a 90 min exposure to M9 buffer for oxidative stress reporter genes (n = 3 independent trials). Note the different y-axis scales between figure panels; scales presented are the same as in Additional file 1 for the respective genes to allow for a direct comparison between N2 and unc-54 mutants. We calculated relative expression by normalization to reference genes followed by normalization to the time point before exercise. We used paired two-tailed Student’s t tests to compare relative expression of control versus M9 buffer samples at each time point. *P < 0.05. (PDF 217 kb) [file 12915_2017_368_MOESM2_ESM.pdf]

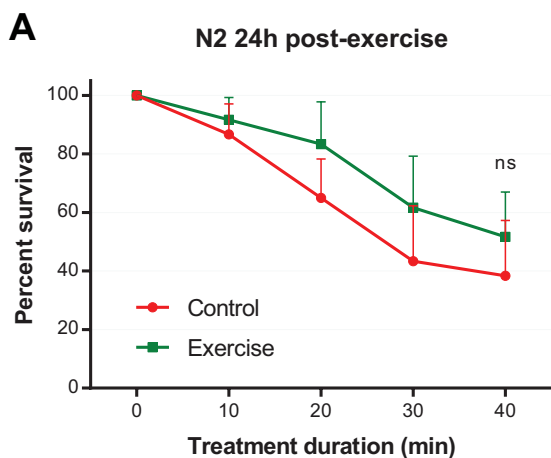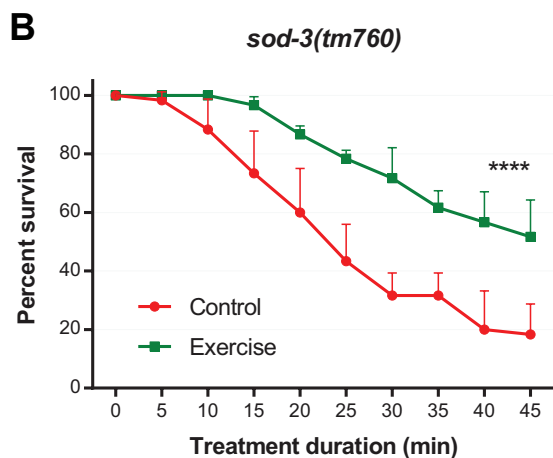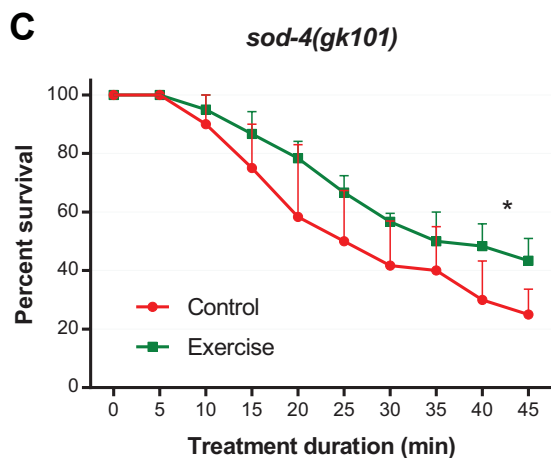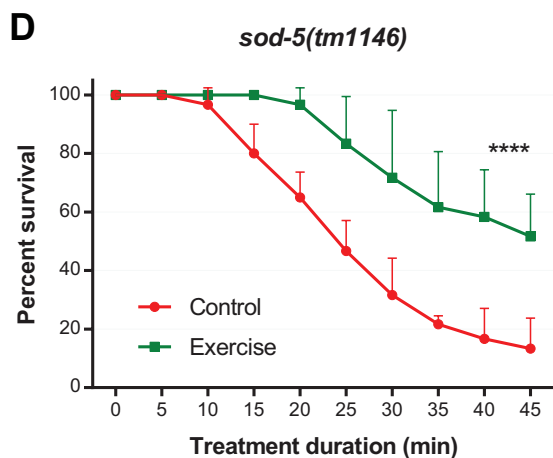

Supplement: Supplementary file 3 — Post-exercise increased survival to juglone treatment is partially dependent on sod-4. (A) Percentage of surviving N2 animals during treatment with 4 mM juglone 24 hours post-exercise (n = 60 animals). Exercised animals still exhibited a trend for increased survival 24 hours post-exercise, although this was statistically non-significant (P = 0.067). We used a higher concentration of juglone for this particular experiment given an increased resistance to juglone of older N2 animals. Percentage of surviving (B) sod-3(tm760), (C) sod-4(gk101), and (D) sod-5(tm1146) animals during treatment with 3 mM juglone 4 hours post-exercise (n = 60 animals). Statistical significance determined by log-rank test. *P < 0.05; ****P < 0.0001. ns non-significant. (PDF 233 kb) [file 12915_2017_368_MOESM3_ESM.pdf]

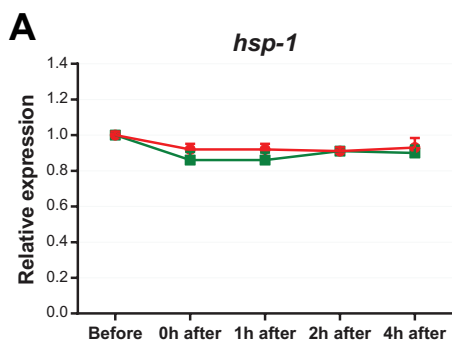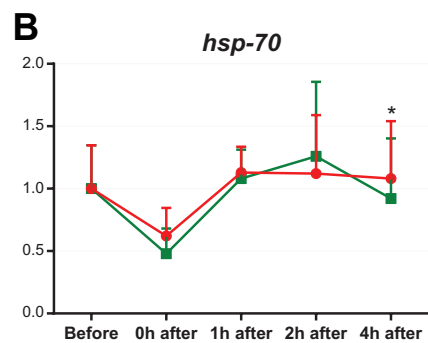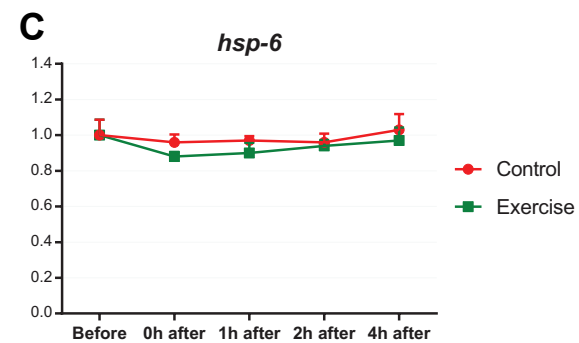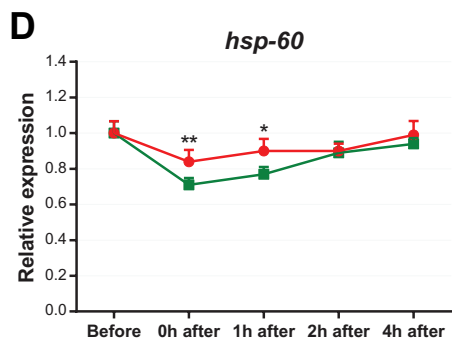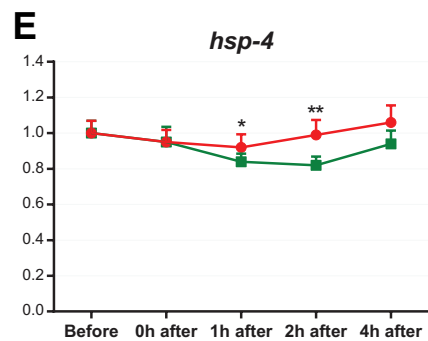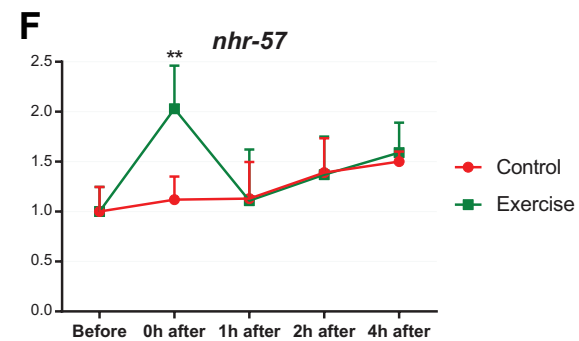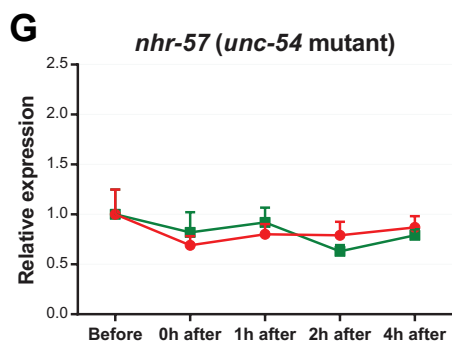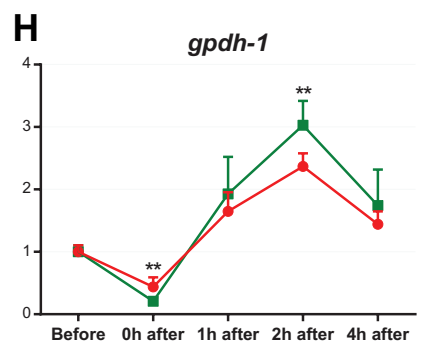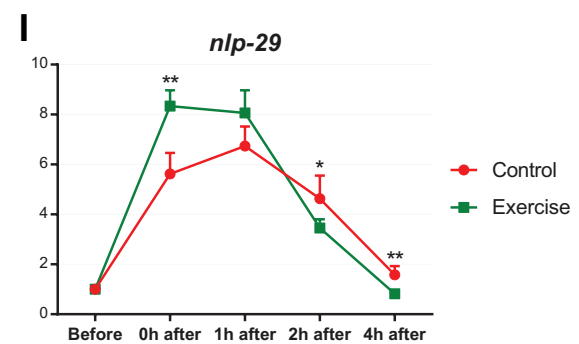

Supplement: Supplementary file 4 — Swim exercise does not induce a generalized stress response in C. elegans. qPCR results before and at different time points post-exercise for (A, B) heat shock response, (C, D) mitochondrial unfolded protein response, (E) endoplasmic reticulum unfolded protein response, (F, G) hypoxia response, and (H, I) osmotic response reporter genes. All panels refer to N2 animals (n = 5 independent trials), except (G), which reports for unc-54 mutants (n = 3 independent trials). Note the different y-axis scales between figure panels; scales presented in (F) and (G) are the same to allow for a direct comparison between N2 and unc-54 mutants. We calculated relative expression by normalization to reference genes followed by normalization to the time point before exercise. We used paired two-tailed Student’s t tests to compare relative expression of control versus exercise samples at each time point. *P < 0.05; **P < 0.01. (PDF 241 kb) [file 12915_2017_368_MOESM4_ESM.pdf]

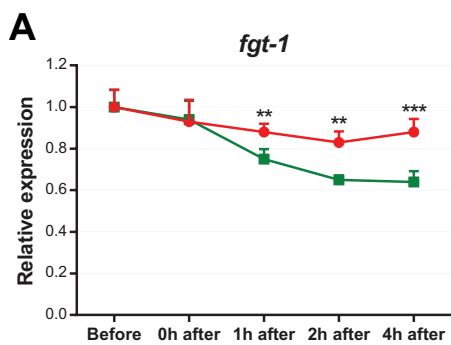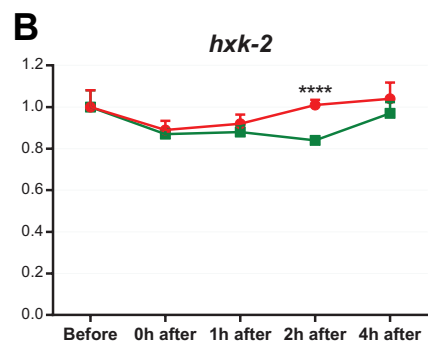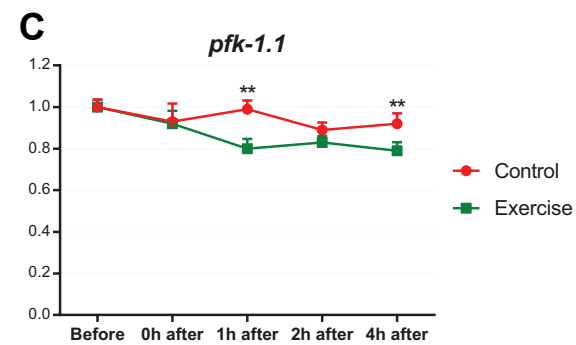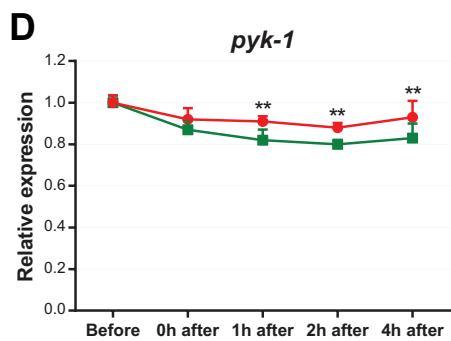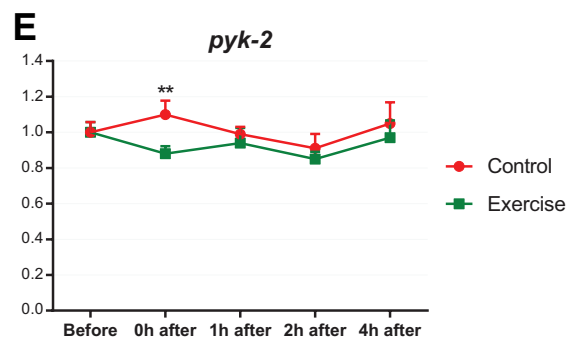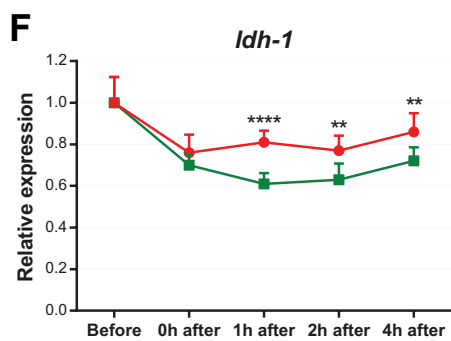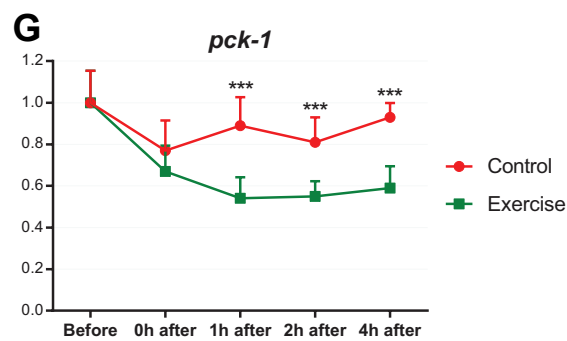

Supplement: Supplementary file 5 — Expression changes are consistent with reduced glucose metabolism after swim exercise in C. elegans. (A–G) qPCR results in N2 animals before and at different time points post-exercise for glucose metabolic genes (n = 5 independent trials). Note the different y-axis scale in (E) compared to all other figure panels. We calculated relative expression by normalization to reference genes followed by normalization to the time point before exercise. We used paired two-tailed Student’s t tests to compare relative expression of control versus exercise samples at each time point. **P < 0.01; ***P < 0.001; ****P < 0.0001. (PDF 227 kb) [file 12915_2017_368_MOESM5_ESM.pdf]

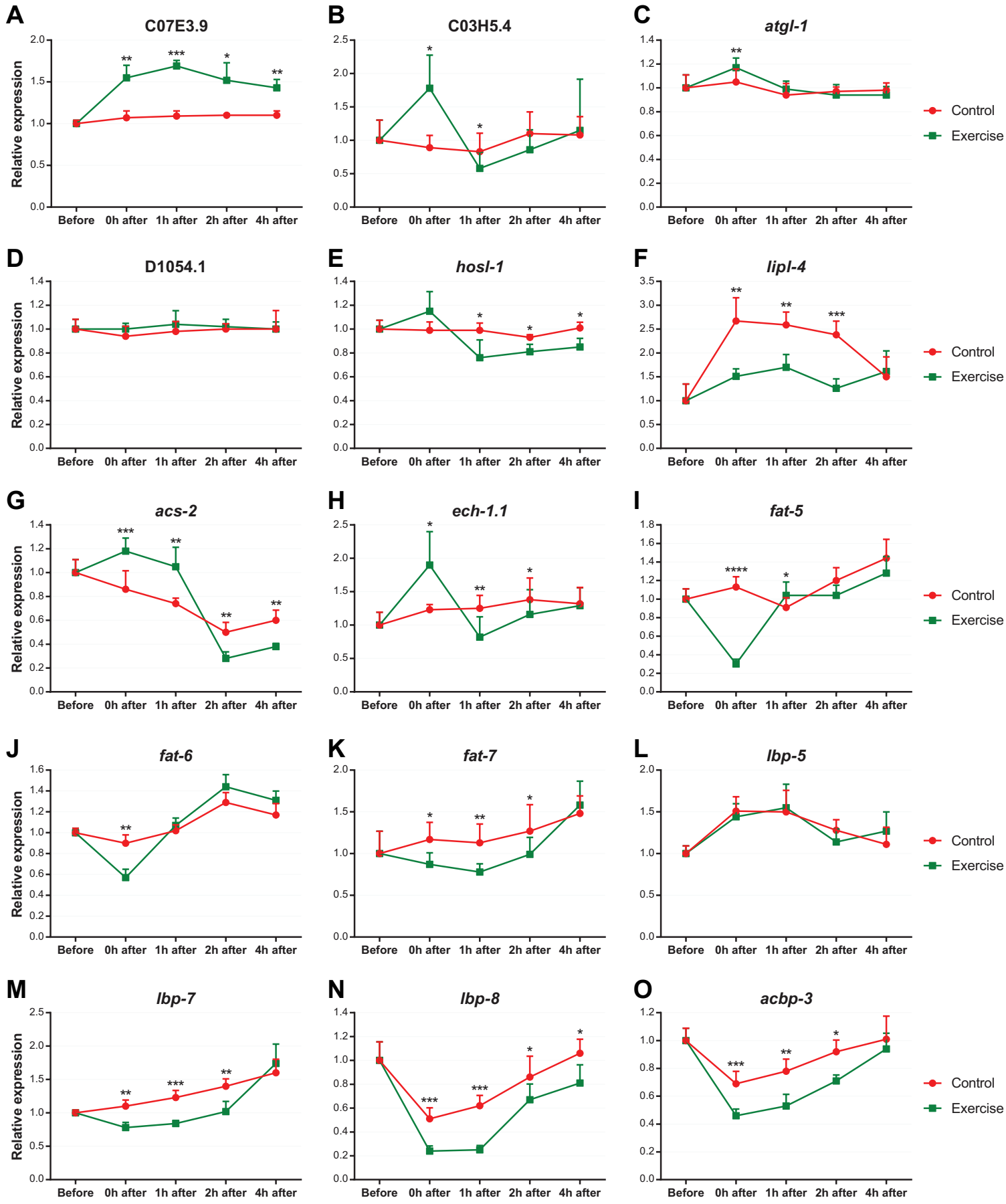

Supplement: Supplementary file 6 — Expression changes are consistent with increased fat metabolism during swim exercise in C. elegans. (A–O) qPCR results in N2 animals before and at different time points post-exercise for fat metabolic genes (n = 5 independent trials). Note the different y-axis scales between figure panels. We calculated relative expression by normalization to reference genes followed by normalization to the time point before exercise. We used paired two-tailed Student’s t tests to compare relative expression of control versus exercise samples at each time point. *P < 0.05; **P < 0.01; ***P < 0.001; ****P < 0.0001. (PDF 269 kb) [file 12915_2017_368_MOESM6_ESM.pdf]
