# Supplementary material for: Single swim sessions in C. elegans induce key features of mammalian exercise
Source: BMC Biol. 2017 Apr 10;15:30. doi: 10.1186/s12915-017-0368-4 (PMC5385602; doi:10.1186/s12915-017-0368-4)
Supplement: Supplementary file 7 — Primers used for qPCR. (DOCX 16 kb) [file 12915_2017_368_MOESM7_ESM.docx]

**Additional file 7. Primers used for qPCR.**

| **Gene** | **Forward primer (5'-3')** | **Reverse primer (5'-3')** | **Amplification**  **efficiency** |
| --- | --- | --- | --- |
| ***acbp-3*** | TTGACAAAATCGCCGAGGAAC | ACTTTCCAATGAGAGCGAGGT | 0.98 |
| ***acs-2*** | GCCAGGAGTTGGACACATCA | TCAGAGTAGACCCAGGCTCC | 1.03 |
| ***atgl-1*** | TGTTTACCACGCTGGAGTCG | GATGAGACCACAGGCAACGA | 0.99 |
| ***cdc-42*** | CTGCTGGACAGGAAGATTACG | CTCGGACATTCTCGAATGAAG | 1.03 |
| ***ctl-1*** | GTGTCGTTCATGCCAAGGGA | GTGGTGTCTGTTTTCCGACC | 1.05 |
| ***ctl-2*** | CTACAGTCGGTGGTGAGAGC | GATCCCAGTTTCCCTCCTCG | 1.04 |
| ***ctl-3*** | AAAGGTGGTGGTGCTCATGG | TGGTGTCTGTTTTCCGACCT | 1.02 |
| **C07E3.9** | CCGAGACTGAAGGCTCTCTG | AGATCCACCGATTCCACACC | 1.02 |
| **C03H5.4** | GTTTTGGGGTTTTTGCTGCC | ACAATAACACCCGTAGCCGT | 1.00 |
| **D1054.1** | TTGTCGTTTAGTGGTTCGGGA | TGCAGATGCACCAGCAAATC | 1.01 |
| ***ech-1.1*** | ATCGAGCTGAGCCAGAAACC | ACAGCAATGCGGTAGTGACA | 0.97 |
| ***fat-5*** | TACCTCCCCTTGGTCGGAAT | AAGCAGTAACGGAAGAGGGC | 1.05 |
| ***fat-6*** | GGGATGGCTTCTTGTGCGTA | GGAAGACGAGAACTGGGTCA | 1.02 |
| ***fat-7*** | ACAAGGCAACCACACCAATG | GTCCACTTGTGATGGCAACG | 1.01 |
| ***fgt-1*** | GTTGTGCCCAGAGTCTCCAA | TCGGTGTCACGGAGCTTTTT | 0.94 |
| ***gcs-1*** | AGGTGAATGCGATGCTTGGA | CGATGAGACCTCCGTAAGGC | 1.02 |
| ***gpdh-1*** | AAACTGGGGATCAGCCATCG | GCGAGAGGTCTCCTGGTTTC | 1.02 |
| ***gst-4*** | TTTGATGCTCGTGCTCTTGC | GGAGTCGTTGGCTTCAGCTT | 0.98 |
| ***hosl-1*** | CGGAGGCGGTTATGTAGCAA | CACAGAGACGACTGGGCAAT | 1.06 |
| ***hsp-1*** | TCTTGACGTTGCCCCACTTT | AGGTTGTGAAGGTCTGAGCG | 0.92 |
| ***hsp-4*** | GCTCGGAAGCAACCAAGATG | TTGTTCTCCTTGTCCTCCCG | 0.95 |
| ***hsp-6*** | TGAAGCTCACCAGAGCCAAG | GGCGATTTGGGAGGACTTGA | 0.92 |
| ***hsp-16.2*** | GATGTTGGTGCAGTTGCTTCG | TTCTCTTCGACGATTGCCTGT | 0.92 |
| ***hsp-16.41*** | TCCGTATTGGAGAAATGCTGA | AGAGACATCGAGTTGAACCGAAA | 0.99 |
| ***hsp-60*** | GGGCGTGGAGATCAAACAGA | AACTTGGCGAGACGCTCATT | 0.96 |
| ***hsp-70*** | CGAACTCCGAAGGGAACAAGA | CGGATTACGAGCGGCTTGAT | 1.07 |
| ***hxk-2*** | GTGCCAGAATTGCCGAATGG | GGGCTCCAGTTCCATCAACA | 0.93 |
| ***lbp-5*** | GCGTGTGCTGCAAAACCAA | TCGTCGAATTCCACTCCCAG | 1.01 |
| ***lbp-7*** | TGGAAGTTGGTCCAAACCGA | GCTTCAAATGAGCAGCAGCC | 1.02 |
| ***lbp-8*** | AGCGATCCTACAACACTTTGGT | CAGTTTTCCGTTTTCGAGCCA | 1.05 |
| ***ldh-1*** | TTCCAGTGTTGCTGTGTGGT | CCTCCCAGTGTTCGTTGTCA | 0.84 |
| **Gene** | **Forward primer (5'-3')** | **Reverse primer (5'-3')** | **Amplification**  **efficiency** |
| ***lipl-4*** | GCTCATCGAGCAATGTGATGG | GAGGAGGTAGCTTTTGCCCA | 0.98 |
| ***nhr-57*** | TCCGACTCTGTGTGGAGTGA | CCCCGTAGTTTGTGGCTCTT | 1.00 |
| ***nlp-29*** | GCTGCCAGCGCACAATG | ACTTTCCCCATCCTCCATACAT | 1.03 |
| ***pck-1*** | TGGAACCATCAACCTGGACG | GGGTGTCGAGGAAGTGTCTG | 1.02 |
| ***pfk-1.1*** | TATGGTCCGTGTGCCACTTC | TCTCTGGAAACTGCGTCCAC | 1.02 |
| ***pyk-1*** | CAAAGAAGCCGAAGCAGCAG | AGATGTGGCAGCGATAGCAA | 1.06 |
| ***pyk-2*** | ATGTGCAACCCAGATGCTCG | CCATCCAAGACAGCGTTTGC | 1.01 |
| ***sod-1*** | TCCGTCACGTAGGCGATCTA | AACGACAGTGTTTGGACCGT | 1.05 |
| ***sod-2*** | GCTCTTCAGCCAGCTCTCAA | TGGTTCTCCTCCGTCCTTTG | 1.04 |
| ***sod-3*** | GGGAGCACGCCTACTACTTG | AGCATTGGCAAATCTCTCGC | 0.98 |
| ***sod-4*** | TGCACCAGATGACTCGAACA | TGAGGCAAGAGAGTCGGAAAC | 0.95 |
| ***sod-5*** | AAGCCGAAGGTGAAGAGACC | GCAGACGTACATCCATCGGT | 0.95 |
| **Y45F10D.4** | GTCGCTTCAAATCAGTTCAGC | GTTCTTGTCAAGTGATCCGACA | 0.94 |
